# Fear of Happiness Predicts Concurrent but not Prospective Depressive Symptoms in Adolescents

**DOI:** 10.32872/cpe.10495

**Published:** 2023-06-29

**Authors:** Merle Kock, Eline Belmans, Filip Raes

**Affiliations:** 1 Centre for the Psychology of Learning and Experimental Psychopathology, KU Leuven, Leuven, Belgium; 2 Child & Youth Institute, KU Leuven, Leuven, Belgium; 3 Leuven Mindfulness Centre, KU Leuven, Leuven, Belgium; Philipps-University of Marburg, Marburg, Germany

**Keywords:** adolescents, dampening, depression, fear of happiness, positive affect, anhedonia

## Abstract

**Background:**

It is increasingly recognised that the study of responses to positive emotions significantly contributes to our understanding of psychopathology. Notably, positive emotions are not necessarily experienced as pleasurable. Instead, some believe that experiencing happiness may have negative consequences, referred to as fear of happiness (FOH), or they experience a fear of losing control over positive emotions (FOLC). According to reward devaluation theory, such an association of positivity with negative outcomes will result in positive stimuli being devalued over time, contributing to or maintaining depressive symptoms. The prospective relationship between fears of positivity and depressive symptoms is yet to be examined in adolescents. The present longitudinal study investigated whether FOH and FOLC prospectively predict depressive symptoms.

**Method:**

128 adolescents between 16-18 years of age (M = 16.87, SD = 0.80) recruited from two secondary schools in Flanders, Belgium, completed measures of depressive symptoms (Depression Anxiety Stress Scales) including consummatory anhedonia, FOH (Fear of Happiness Scale), and FOLC (Affective Control Scale) in their classroom at baseline and 2-months follow-up. Regression analyses were performed to test the association between FOH, FOLC, and depressive symptoms.

**Results:**

FOH concurrently, but not prospectively, predicted depressive symptoms. There was no significant association between FOH and consummatory anhedonia. FOLC was not a significant predictor of depressive symptoms or consummatory anhedonia.

**Conclusion:**

These findings suggest that FOH may only be concurrently related to depressive symptoms. Considering prior findings in adults, future research should investigate the association of FOH with anticipatory anhedonia in adolescents.

## Highlights

- Concurrent and prospective associations between fears of positivity and adolescents’ depressive symptoms were tested.
- Fear of happiness was concurrently but not prospectively associated with depressive symptoms.
- Fear of happiness did not predict consummatory anhedonia; anticipatory anhedonia was not assessed.
- Fear of losing control over positive emotions did not predict depressive symptoms or anhedonia.

The ability to regulate emotional experience plays a vital role in development and maintenance of emotional disorders in adolescents (Young et al., 2019). Research into emotion regulation has to date primarily focused on negative emotions but it is increasingly recognised that studying positive emotions is also of great value. Because positive and negative emotions are independent of each other, emotion regulation may function differently in each domain (Wood et al., 2003). Moreover, deficits in experience and regulation of positive emotions are present across various forms of psychopathology (Dillon & Pizzagalli, 2010). From a clinical perspective, most psychological treatments are targeting negative emotions and are often ineffective for improving deficits in positive emotion regulation (Dunn, 2012). Thus, investigating positive emotion regulation may contribute to our understanding of psychopathology, particularly depressive disorders, over and above insights gained through research into negative emotion regulation.

## Defining FOH and FOLC

Notably, positive emotions are not necessarily experienced as pleasurable. Instead, empirical evidence suggests that some individuals are even afraid of positive emotions. One reason may be the belief that experiencing happiness may have negative consequences, referred to as fear of happiness (FOH; Joshanloo, 2013). Individuals may experience FOH because they are more afraid of the loss after feelings of happiness have ended than they value experiencing feelings of happiness. Other individuals experience FOH because they have repeatedly been disappointed when looking forward to pleasurable activities and are afraid of being disappointed again. Another reason for fearing positive emotions may be that individuals are afraid of losing control over their positive emotions (FOLC; Williams et al., 1997), for example because they get carried away with their excitement and consequently become careless.

Generally, deficits in the experience of positive emotions predict a poor prognosis of depression (Morris et al., 2009), possibly because positive emotions were found to increase resilience against negative life events (Tugade & Fredrickson, 2004). However, fear of positive emotions may prevent individuals from savouring positive emotions and using them to cope with adversities. For example, a patient with an agoraphobic mother reported getting excited to go to the beach as a child, which repeatedly ended in her mother experiencing a panic attack, triggering an argument with her father, and creating a terrible atmosphere. As a result, the patient felt she would be better off not looking forward to enjoyable activities because she got to associate positive emotions with negative outcomes (P. Gilbert, 2007). According to reward devaluation theory, such a repeated association of positive emotions with either an ultimate negative outcome or simultaneous negative emotions may result in positive stimuli being devalued over time (Winer & Salem, 2016). Positive stimuli are consciously inhibited or avoided because individuals fear that their initially positive experience will result in negative outcomes. Ultimately, positivity becomes a signal of negative affect (Jordan et al., 2021), which may be reflected in FOH. A meta-analysis (Winer & Salem, 2016) provides evidence for reward devaluation theory by showing that depressed patients are more likely to avoid positive information in a dot probe task compared to anxious patients and healthy controls. Moreover, two experimental studies demonstrated that pairing environmental reward with inhibition of rewarding behaviour slowed responses to reward or reduced the reward value (Veling et al., 2011; Veling & Aarts, 2009). Notably, inhibition of reward was only visible in participants initially sensitive to the reward, suggesting the initially-rewarding stimulus was devalued rather than lacked value from the start.

Because FOH is characterised by deficits in the positive affect system, it may be specifically related to anhedonia, a hallmark symptom of depression. Anhedonia encompasses both deficits in looking forward to pleasurable events (anticipatory anhedonia) and deficits in experiencing pleasure during an enjoyable event (consummatory anhedonia) (Gard et al., 2006). Since individuals with FOH associate happiness with negative consequences, they may lack motivation to approach pleasurable events and may in turn develop anticipatory anhedonia. Ultimately, this increase in anticipatory anhedonia may contribute to the development of other symptoms of depression such as sadness and lack of hope because individuals lack motivation to approach reward. This was supported by Jordan et al. (2018) who found anticipatory anhedonia to mediate the relationship between fear of positive evaluation, another fear of positivity related to FOH, and other depressive symptoms in adults. On the other hand, individuals with FOH may also experience consummatory anhedonia when confronted with positive events because they associate positivity with negative outcomes. This may trigger other depressive symptoms such as lack of hope or sadness when they realise that they cannot enjoy positive experiences anymore. For adolescents, who cannot withdraw as easily when caregivers confront them with pleasurable experiences, this may be especially relevant. Hence, FOH may be associated with and predict anticipatory and consummatory anhedonia, which in turn contributes to other depressive symptoms. Previous research found that FOH is strongly correlated with depression, anxiety, and stress (P. Gilbert et al., 2012). Using a slightly different measure of FOH, Joshanloo et al. (2014) showed that FOH predicted lower life satisfaction above a set of recognized predictors at the individual (e.g., autonomy) and cultural level (e.g., wealth). These findings of cross-sectional studies demonstrate that FOH is associated with lower wellbeing and psychopathology. There is currently only one study providing evidence for a significant positive prospective link between FOH and depressive symptoms in adults (Jordan et al., 2021).

In contrast to FOH, FOLC reflects losing control over positive emotions and may therefore be more related to bipolar disorder. Given FOLC’s effect on the positive valence system, it may be especially associated with anhedonia. Individuals with FOLC may be unable to look forward to pleasurable events (anticipatory anhedonia) because they anticipate losing control of their emotions, but they may also be unable to enjoy pleasurable events in the moment (consummatory anhedonia) because they fear to lose control any moment instead of enjoying the experience. This feeling of lack of control may be especially prominent in adolescents as affective control is reduced during adolescence compared to childhood and adulthood (Schweizer et al., 2020). Notably, poor affective control is associated with mental health problems. Also fear of losing affective control (i.e. FOLC) has been associated with increased depressive symptoms (Yoon et al., 2018). Yet, findings are limited by the cross-sectional design of previous studies and FOLC’s influence on depressive symptoms requires further investigation.

## Importance of Assessing an Adolescent Sample

Adolescence is a crucial period with regard to mental health because a substantial amount of depressed patients experience their first episode in adolescence (Zisook et al., 2007). Given the possible role of FOH and FOLC in the development of depressive disorders, it is important to study the associations of FOH and FOLC with depressive symptoms not only in adults, which has been done in prior research, but also in adolescents. Understanding which factors contribute to the development of depressive symptoms in adolescence would allow us to counteract the alarming rise of mental disorders among young people (Patel et al., 2007). This rise is to be expected considering that adolescents undergo an emotionally challenging period, in which they develop strategies to regulate their emotions more independently. However, research on the use, adaptiveness, and effectiveness of emotion regulation strategies in adolescents is scarce (Riediger & Klipker, 2014). Two experimental studies found that inducing thoughts to downregulate positive emotions (dampening) completely reduced the effects of a positive memory recall in adults while in adolescents the positive memory still positively impacted happiness (Dunn et al., 2018; Yilmaz et al., 2019). These findings support the idea that appraisal-based emotion regulation strategies like dampening are less potent in adolescents because top-down cognitive control is still developing (Skinner & Zimmer-Gembeck, 2016). In sum, adolescence is an important period for emotional development. Given that emotion regulation strategies, or at least their effects, seem to differ between adults and adolescents it is important to better understand how adolescents respond to emotions in order to counteract the alarming rise in mental disorders.

## The Present Study

This study aims to investigate whether FOH and FOLC prospectively predict depressive symptoms. 128 adolescents completed self-report questionnaires of depressive symptoms (including consummatory anhedonia), FOH, and FOLC at baseline and 2-months later. Based on prior cross-sectional research, we hypothesized that FOH and FOLC would cross-sectionally and prospectively predict depressive symptoms including anhedonia. Hypotheses were formulated prior to data analysis.

## Method

### Participants

Our sample was recruited as part of a larger study aiming to test whether negative self-referent processing predicts depressive symptoms in adolescents (Belmans et al., 2023). For this larger study, a power analysis in G*Power (Faul et al., 2007) indicated a required sample of *N* = 58 participants to reach a power of .80 with α = .05 based on a cross-sectional effect size of Cohen’s *d* = .82 (Iijima et al., 2017). The larger study oversampled to account for attrition and because smaller prospective effects were expected compared to previously observed cross-sectional effects. School classes, rather than individual participants, were recruited from two secondary schools in Flanders, Belgium, resulting in a total sample of 128 adolescents (60.63% female). Adolescents were 16-18 years old (*M* = 16.87, *SD* = 0.80) and most were of Belgian origin (80%). At follow-up assessment, 11 adolescents (8.7%) did not participate because they were absent from school on the day of assessment.

The age group was chosen to ensure that participants understand the computer task in the larger study. Sensitivity analyses conducted in G*Power revealed that the present study was able to detect a small-to-medium effect (Cohen’s *f* = .28) in concurrent and prospective multiple regression models given *N* = 128, a power of .80, and α = .05. The study was approved by the Social and Societal Ethics Committee at KU Leuven (G-2018-01-1090) and all participants provided informed consent in accordance with the Declaration of Helsinki (World Medical Association, 2013).

### Measures

#### Depression Subscale of Depressive Anxiety Stress Scales (DASS-D)

Depressive symptoms were assessed with the 7-item DASS-D (Lovibond & Lovibond, 1995). Participants indicated on a 4-point scale, from d*id not apply to me at all* to *applied to me very much, or most of the time*, how they felt during the past week (e.g., *I felt down-hearted and blue*). One item assesses consummatory anhedonia (*I couldn’t seem to get any enjoyment out of the things I did*). The total score is calculated as the sum of all item scores. The Dutch DASS-D has good psychometric properties (de Beurs et al., 2001).

#### Fear of Happiness Scale (FOHS)

To assess fear of happiness, the Dutch FOHS was used (Joshanloo, 2013; Nelis et al., n.d.). Its 5 items are scored on a 7-point scale ranging from *strongly disagree* to *strongly agree* (e.g., *I prefer not to be too joyful, because usually joy is followed by sadness*).

#### Positive Affect Subscale of Affective Control Scale (ACS-PA)

FOLC was assessed with the 13-item ACS-PA (Raes et al., 2017; Williams et al., 1997). On a 7-point scale ranging from *very strongly disagree* to *very strongly agree*, participants indicated how they respond to positive affect (e.g., *When I feel really happy, I go overboard, so I don’t like getting overly ecstatic*).

### Procedure

At baseline and 2-months follow-up, participants completed all questionnaires and a computer task that is not part of this study collectively in their classrooms. The duration of follow-up was chosen such that both assessments took place in the same school year to minimise attrition.

### Statistical Analyses

To test whether FOH and FOLC predicted concurrent and prospective depressive symptoms, regression analyses with DASS-D scores as criterion variable were performed for cross-sectional and prospective data separately. FOH and FOLC scores were entered as predictors and the dummy-coded variable female was added as covariate. For prospective analyses, DASS-D scores at baseline were entered as in a first step, before all other predictors were entered. Since previous studies identified anhedonia as a mediator between fear of positive evaluation and depressive symptoms, we performed post-hoc analyses to test the association between fears of positivity and the single-item measure of consummatory anhedonia from the DASS-D scale (Item 1). Using this item as criterion variable, we conducted an additional ordinal logistic regression. Predictor variables were the same as in aforementioned analyses except for prospective analyses, in which the baseline consummatory anhedonia score was entered in the first step. *z*-scores of continuous predictors were added to compute standardised odds ratios as a measure of effect size. Collinearity between predictors was assessed by a Variance Inflation Factor (VIF) larger than 10. To confirm that the proportional odds assumption was met, the brant test was applied (Brant, 1990). Additionally, the proportional odds assumption for each predictor was checked using likelihood ratio tests comparing a proportional odds model with a partial proportional odds model for which the proportional odds assumption was relaxed for the respective predictor.

Benjamini-Hochberg adjustment for multiple testing was applied to all *p*-values except those testing a priori hypotheses. We reported partial *R*^2^ as effect size with .02, .13, and .26 indicating small, medium, and large effects, respectively (Cohen, 1992). For the ordinal regression analysis, we reported *OR* as effect size with 1.44, 2.48, and 4.27 indicating small, medium, and large effects, respectively (Sánchez-Meca et al., 2003). Missing data was limited. 11 participants were lost to follow-up because they were not present at school on the day of assessment. Only their baseline data was included in the analysis. Additionally, single items were missing from the DASS-D and FOLC scales for individual participants. In total, there were 0.002% of DASS-D items missing at baseline, 0.004% of FOLC items missing at baseline, and 0.0007% of FOLC items missing at follow-up. Little’s test for MCAR demonstrated that missing data at both time points were missing completely at random (Little, 1988). Missing items were imputed using the mean score of all remaining questionnaire items. Analyses were conducted in R (R Core Team, 2021) using the stats package (version 4.1.1) for linear regression analyses and the VGAM package (version 1.1-7) for ordinal regression analyses (Yee, 2022).

## Results

### Descriptive Statistics and Internal Consistency

Means, standard deviations, ranges, and Cronbach’s α for all measures are reported in Table 1.

**Table 1:** Descriptive Information for Baseline and Follow-up Measures

Variable	*n*	*M*	*SD*	Min	Max	α
Assessment T1						
DASS-D	127	4.39	3.98	0	17	.84
FOH	127	13.69	6.85	5	35	.89
FOLC	127	39.38	9.54	15	60	.82
Assessment T2						
DASS-D T2	116	3.92	3.68	0	15	.83
FOH T2	116	12.21	6.34	5	28	.89
FOLC T2	116	37.20	10.33	13	60	.83

*Note.* α = Cronbach’s alpha.

### Correlational Analyses

Zero-order Pearson correlations revealed significant correlations of depressive symptoms with FOH and FOLC at baseline (Table 2). Higher levels of depressive symptoms were associated with greater FOH and FOLC. Zero-order correlations between predictors at baseline and depressive symptoms at follow-up yielded similar results.

**Table 2:** Pearson Correlations Between Depressive Symptoms (DASS-D), Fear of Happiness, and Fear of Losing Control Over Positive Emotions

Variable	1	2	3	4	5	6
1. DASS-D	‒	.45***	.26**	.69***	.37***	.27**
2. FOH	[.30, .58]	‒	.50***	.36***	.69***	.39***
3. FOLC	[.09, .42]	[.35, .62]	‒	.25**	.41***	.68***
4. DASS-D T2	[.58, .77]	[.19, .51]	[.07, .41]	‒	.45***	.34***
5. FOH T2	[.20, .52]	[.58, .78]	[.25, .55]	[.29, .58]	‒	.52***
6. FOLC T2	[.09, .43]	[.23, .54]	[.57, .77]	[.17, .49]	[.38, .64]	‒

*Note.* Pearson correlations with Benjamini-Hochberg adjustment for multiple testing are reported above the diagonal. 95% confidence intervals are reported below the diagonal.

*p < .05. **p < .01. ***p < .001.

### Regression Analyses

Results of regression analyses are displayed in Table 3 and will be reported using effect sizes and corresponding confidence intervals (CIs). An effect size of zero indicated that the predictor did not significantly impact the outcome. Hence, when a CI does not include zero, the effect is considered significant.

**Table 3:** Summary of Regression Analyses for Variables Predicting Depressive Symptoms (DASS-D) at T1 and T2

Variable	*B* (*SE*)	*B* 95% CI	β	*p*	partial *R*^2^	*R* ^2^	VIF
DV: DASS-D T1							
Constant	0.04 (1.40)	[-2.72, 2.81]		.97			
Female	0.21 (0.65)	[-1.08, 1.51]	.03	.74	.001 [0, .03]		1.01
FOH T1	0.25 (0.05)	[0.14, 0.35]	.43	< .001	.140 [.05, .26]		1.34
FOLC T1	0.02 (0.04)	[-0.06, 0.10]	.05	.59	.002 [0, .03]	.21	1.33
DV: DASS-D T2							
Step 1							
Constant	0.90 (0.47)	[-0.03, 1.83]		.06			
Female	0.30 (0.52)	[-0.73, 1.33]	.04	.56	.002 [0, .03]		1.02
DASS-D T1	0.66 (0.07)	[0.53, 0.80]	.68	< .001	.460 [.28, .61]	.48	1.02
Step 2							
Constant	-0.19 (1.08)	[-2.33, 1.95]		.86			
Female	0.25 (0.52)	[-0.79, 1.28]	.03	.64	.001 [0, .03]		1.04
DASS-D T1	0.62 (0.07)	[0.48, 0.77]	.64	< .001	.340 [.18, .51]		1.20
FOH T1	0.04 (0.05)	[-0.05, 0.13]	.07	.37	.004 [0, .04]		1.49
FOLC T1	0.02 (0.03)	[-0.04, 0.08]	.05	.52	.002 [0, .03]	.49	1.33

*Note.* 95% percentile bootstrap confidence intervals for partial *R*^2^ are reported in brackets.

FOH was significantly associated with depressive symptoms at baseline with a medium effect size (partial *R*^2^ = .14, 95% CI [.05, .26]), with greater FOH predicting higher levels of depressive symptoms. FOH did not significantly predict depressive symptoms at follow-up when controlling for depressive symptoms at baseline, which is reflected in the effect size falling below the cut-off for a small effect (partial *R*^2^ = .004, 95% CI [0, .04]). However, FOH significantly predicted depressive symptoms at follow-up with a small-to-medium effect size when baseline depressive symptoms were deleted from the model (partial *R*^2^ = .07, 95% CI [.004, .19]; see Appendix A in the Supplementary Materials). FOLC was not significantly associated with depressive symptoms at baseline nor at follow-up. The effect size for both concurrent and prospective associations of FOLC with depressive symptoms fell well below the threshold for a small effect (see Table 3). An examination of VIFs confirmed no violations of multicollinearity (see Table 3).

Ordinal logistic regression analyses using the single-item anhedonia score as criterion variable are displayed in Table 4 and will be reported using odds ratios (*OR*) and corresponding CIs. An *OR* of one indicated that there is no association between predictor and outcome. Hence, when a CI does not include one, the effect is considered significant. Due to low frequencies of the outcome categories “Applied to me to a considerable degree or a good part of time” and “Applied to me very much or most of the time” for consummatory anhedonia, these two categories were combined to increase statistical power of the overall model. For the model predicting anhedonia at baseline, the proportional odds assumption for FOLC was violated and a partial proportional odds model was used instead. For all other predictors in both models, the proportional odds assumption was satisfied.

**Table 4:** Summary of Ordinal Logistic Regression Analyses for Variables Predicting Consummatory Anhedonia (Single Item DASS-D) at T1 and T2

Variable	β (*SE*)	*p*	*OR*	*OR* 95% CI	VIF
DV: Anhedonia T1					
Female	0.38 (0.36)	.71	1.46	[0.73, 2.93]	1.01
FOH T1	0.47 (0.21)	.06	1.61	[1.07, 2.41]	1.34
Comparison: (Applied to a considerable degree & Applied to some degree) vs. Did not apply at all					
FOLC T1	-0.01 (0.21)	.97	0.99	[0.66, 1.49]	1.33
Comparison: Applied to a considerable degree vs. (Applied to some degree & Did not apply at all)					
FOLC T1	-0.60 (0.30)	.29	0.55	[0.31, 0.99]	
Nagelkerke Pseudo-*R*^2^ = 0.09					
DV: Anhedonia T2					
Female	0.15 (0.39)	.71	1.16	[0.54, 2.50]	1.04
Anhedonia at T1 [not at all as reference]					
To some degree	1.11 (0.41)	.01	3.02	[1.34, 6.80]	1.03
To a considerable degree	2.01 (0.65)	.01	7.46	[2.08, 26.81]	
Fears of positive emotions					
FOH T1	0.44 (0.22)	.07	1.56	[1.01, 2.41]	1.37
FOLC T1	0.22 (0.22)	.64	1.24	[0.81, 1.91]	1.33

*Note.* Nagelkerke Pseudo-*R*^2^ = 0.23.

After multiple testing correction, there was a trend towards an association between FOH and consummatory anhedonia at baseline (*OR* = 1.61; 95% CI [1.07, 2.41]), meaning that a one unit increase in FOH at baseline was associated with a 61% increase in the odds to experience consummatory anhedonia at baseline to some or a considerable degree as compared to not at all. Similarly, there was a trend towards an association between FOH at baseline and consummatory anhedonia at follow-up when controlling for consummatory anhedonia at baseline (*OR* = 1.56; 95% CI [1.01, 2.41]), meaning that a one unit increase in FOH at baseline was associated with a 56% increase in the odds to experience anhedonia at follow-up to some or a considerable degree as compared to not at all. FOLC was not significantly associated with consummatory anhedonia at baseline nor at follow-up (see Table 4). An examination of VIFs confirmed no violations of multicollinearity (see Table 4).

## Discussion

This study aimed to investigate whether FOH and FOLC concurrently and prospectively predict depressive symptoms in adolescents. Results showed that higher levels of FOH are related to higher concurrent depressive symptoms but were not predictive of depressive symptoms two months later. FOLC was not a significant predictor of depressive symptoms or anhedonia at the concurrent or prospective level. Importantly, it is unlikely that the lack of significant prospective associations with depressive symptoms was caused by low power. A post-hoc sensitivity analysis revealed that the minimum detectable effect size in this study was small-to-medium (*f*^2^ = 0.085) given α = .05 and a power of .80. From a clinical perspective, effects that are smaller than this small-to-medium effect are unlikely to make a meaningful impact in clinical practice as small effects can easily be overshadowed by other influencing factors. Thus, the current study was sufficiently powered to detect an effect that is clinically meaningful. This suggests that the lack of significant prospective associations is not caused by low power but may be explained by a negligible prospective association between FOH, FOLC, and depressive symptoms in our sample.

Our findings are in line with prior research on a closely related construct, i.e. dampening (Feldman et al., 2008; Nelis et al., 2015). Dampening is defined as downgrading positive emotions by decreasing intensity and duration of positive mood states (Feldman et al., 2008). Therefore, dampening can be regarded as a broader concept that partly encompasses the construct of FOH because some dampening thoughts include the fear-related aspect of FOH while other dampening thoughts are not related to FOH. In alignment with our findings, increased dampening has been consistently associated with higher levels of concurrent depressive symptoms in adults and adolescents (Feldman et al., 2008; Nelis et al., 2015). However, results on the prospective association between dampening and depressive symptoms are mixed, with some studies reporting that dampening predicts increased depressive symptoms (Hudson et al., 2015; Raes et al., 2012) and others reporting absence of effects (K. E. Gilbert et al., 2013; Nelis et al., 2015).

Notably, there is some evidence that dampening may be specifically predictive of anhedonia (Nelis et al., 2018). Since anhedonia includes diminished pleasure in positive experiences, it might be more strongly linked to dampening responses compared to general depressive symptoms. Similarly, fear of positive evaluation, another type of fear of positivity closely linked to FOH, has been shown to affect depressive symptoms via anticipatory anhedonia (Jordan et al., 2018). Considering the similarities of, and strong correlation between dampening and FOH, FOH may display similar correlation patterns with anhedonic symptoms compared to general depressive symptoms. In this study, we observed a trend towards a concurrent and prospective association between FOH and consummatory anhedonia but no prospective association between FOH and general depressive symptoms. Moreover, the prospective association between FOH and depressive symptoms decreased when the consummatory anhedonia item was excluded from the measure of depressive symptoms (see Appendix B in the Supplementary Materials). However, the size of the association between FOH and consummatory anhedonia is rather small and did not pass the multiple testing correction. One possible explanation for this non-significant association of FOH with consummatory anhedonia may be the use of a single-item measure. This measure may be problematic because single-item measures are more affected by measurement error as they cannot be compared to corresponding items measuring the same construct, resulting in lower or at least unknown reliability compared to multi-item scales (Allen et al., 2022). Moreover, the used single-item measure only captures consummatory but not anticipatory anhedonia. This is important given that Jordan et al. (2018) found that anticipatory, but not consummatory, anhedonia mediates the effect of fear of positive evaluation on depressive symptoms. It is possible that FOH, like fear of positive evaluation, mainly affects anticipatory and to a lesser extent consummatory anhedonia. Future studies should use a more fine-grained measure of anhedonia to differentiate the relationships between FOH, anticipatory and consummatory anhedonia, and depressive symptoms.

This study was carried out in a non-clinical sample. It is possible that the prospective association between FOH and depressive symptoms is only evident in clinical populations with stronger depressive symptoms at baseline. However, one prior study did not find a prospective association between dampening and depressive symptoms in remitted depressed patients (K. E. Gilbert et al., 2013), suggesting that there is no prospective link between dampening and depressive symptoms in clinically-depressed populations. On the other hand, Jordan et al. (2018) found an effect of fear of positive evaluation on depressive symptoms via anticipatory anhedonia in a community sample with mild depressive symptoms. Future studies should disentangle the relationship between fears of positivity and depressive symptoms in clinical samples.

Unexpectedly, we did not find any association between FOLC and depressive symptoms. One possible explanation is that the original factor structure of the ACS is based on expert opinion and does not provide acceptable fit in factor analyses (Melka et al., 2011). However, re-analysing the data with the factor structure derived from exploratory factor analysis did not change the results (see Appendix C in the Supplementary Materials), suggesting that FOLC has no association with depressive symptoms in adolescents, at least not in our sample.

The main limitations of our study were the reliance on self-report measures and the use of the DASS as only measure of depressive symptoms. The DASS mainly assesses symptoms related to negative emotions and only includes one item measuring consummatory anhedonia. Moreover, the average scores on the DASS-D are quite low in our sample compared to a Dutch-speaking clinically depressed sample (de Beurs et al., 2001). Future studies should investigate the relationship between FOH, FOLC, and depressive symptoms in adolescent samples with more prominent depressive symptoms. Another limitation of this study is the failure to measure positive emotions. Future studies should specifically assess positive emotions to examine whether the association of FOH and depressive symptoms is dependent on the current level of positive emotions.

In conclusion, this study shows that FOH is concurrently but not prospectively associated with depressive symptoms. There was no significant association between FOH and the single-item measure of consummatory anhedonia, however, anticipatory anhedonia was not assessed. In light of prior findings on the effect of related fears of positivity on anticipatory anhedonia in adults, future research should investigate the concurrent and prospective association between FOH and anticipatory anhedonia in adolescents using a more fine-grained measure of anhedonia.

## Supplementary Materials

The Supplementary Materials contain the following items (for access see Index of Supplementary Materials below):

- *Code used for analyses*
- *Appendix A*: Regression Analysis for Variables Predicting Depressive Symptoms (DASS-D) at T2 Without Controlling for Baseline Depressive Symptoms
- *Appendix B*: Hierarchical Regression Analysis for Variables Predicting Depressive Symptoms Excluding the Anhedonia Item (DASS-D 2) at T2
- *Appendix C*: Hierarchical Regression Analyses for Variables Predicting Depressive Symptoms (DASS-D) at T2 Using the Updated Factor Structure of ACS

KockM.
BelmansE.
RaesF.
 (2023a). Supplementary materials to "Fear of happiness predicts concurrent but not prospective depressive symptoms in adolescents"
[Analysis code]. PsychOpen. https://osf.io/r9gkm
10.32872/cpe.10495PMC1050825737732149

KockM.
BelmansE.
RaesF.
 (2023b). Supplementary materials to "Fear of happiness predicts concurrent but not prospective depressive symptoms in adolescents"
[Additional analyses]. PsychOpen. 10.23668/psycharchives.12919
PMC1050825737732149

## Data Availability

The data that support the findings of this study are available on request from the corresponding author. The data are not publicly available due to privacy or ethical restrictions.
